# Association between distinct coping styles and heart rate variability changes to an acute psychosocial stress task

**DOI:** 10.1038/s41598-021-03386-6

**Published:** 2021-12-15

**Authors:** Arthur Viana Machado, Mirtes Garcia Pereira, Gabriela G. L. Souza, Mariana Xavier, Carolina Aguiar, Leticia de Oliveira, Izabela Mocaiber

**Affiliations:** 1 Laboratory of Neurophysiology of Behavior, Physiology and Pharmacology Department, Biomedical Institute, Federal Fluminense University, Niterói, RJ Brazil; 2 Laboratory of Cognitive Psychophysiology, Department of Natural Sciences, Institute of Humanities and Health, Federal Fluminense University, Rio das Ostras, RJ Brazil; 3 Laboratory of Psychophysiology, Department of Biological Sciences, Federal University of Ouro Preto, Ouro Preto, MG Brazil

**Keywords:** Psychology, Physiology

## Abstract

Modern life comprises a myriad of stressful situations, ranging from life-threatening ones to others not so deadly, all of which activate a physiologic stress response. Engaging in healthy ways to cope can prevent us from wearing out our physiological systems. Heart rate variability (HRV) is often used as an index of emotion regulation response. Hence, our goal is to investigate whether the habitual use of coping strategies is related to a distinct pattern of HRV changes when the individual is exposed to a moderate psychosocial stressor. In this study, 60 female participants performed a psychosocial stress task—oral speech preparation—while ECG signals were collected during the whole experimental procedure. Heart rate (HR), HRV parameters (SDNN, RMSSD, LF, HF) and coping strategies (Brief COPE) were registered. Participants were divided into two groups (low and high groups) as a function of their scores on the maladaptive and adaptive coping strategies of the Brief COPE. As expected, the task alone induced increases in heart rate and reductions in HRV parameters. Additionally, the analyses revealed a different pattern of HRV (SDNN, RMSSD, LF and HF) changes in response to the stressor, with participants using less maladaptive strategies being able to maintain the HRV at baseline levels when confronting the stressor, while those using more maladaptive strategies reducing HRV during the task. These results show a different pattern of HRV changes as a function of the coping style, suggesting a possible autonomic advantage, namely, the maintenance of HRV, in individuals who use maladaptive coping strategies less frequently.

## Introduction

The stress response comprises a set of behavioral and physiological responses to challenging situations, marked by the activation of the autonomic nervous system and the hypothalamic–pituitary–adrenal (HPA) axis. From an evolutionary perspective, the stress response is crucial for survival and adaptation to environmental demands. However, the stress response can become harmful to our health when it is inadequate, exaggerated or chronically activated^1^. These demanding and challenging situations disturbing body homeostasis is present in various forms in our modern society, ranging from a life-threatening situation such as an armed assault to a moderately stressful event such as an oral presentation for a class. As Sapolsky said, “It is not a general mammalian trait to become anxious about mortgages or the Internal Revenue Service, about public speaking or fears of what you will say in a job interview, about the inevitability of death”^2^. Like it or not, this ever-changing world we live in today requires us to deal with different situations that often induce a stress response. In this vein, engaging in healthy ways to cope with stress can prevent us from wearing out our physiological systems.

In fact, we can cope with stressors using distinct strategies to minimize distress associated with negative events. Stress and coping theory considers coping to be the person’s cognitive and behavioral efforts to manage internal and external demands in a stressful transaction^3,4^. Folkman and Lazarus classified coping into two types: problem-focused coping, which involves addressing the problem causing distress such as actively coping with or planning how to deal with the problem, and emotion-focused coping, which aims to ameliorate the negative emotions associated with the stressor such as reappraising, seeking emotional support, or meditating^5^. Another common way to categorize coping styles is using the adaptive (i.e., helpful, functional) and maladaptive (i.e., problematic, negative, dysfunctional) nomenclature, since problem-focused and emotion-focused coping can be useful or detrimental depending on the situation or the type of strategy employed to face the stressor^5–8^. According to Carver et al. (1989), a given coping strategy may not be intrinsically maladaptive but may become dysfunctional if it is relied on for long periods when other strategies are more useful. It is important to note that the concept of coping is sometimes related to the construct of emotion regulation, a process by which individuals modify their emotional experiences, expressions, and subsequent physiological responses^9^. In fact, the terms are sometimes used interchangeably without clear limits or distinctions. Eisenberg et al. (1997) classify both coping and emotion regulation as instances of a more general category of self-regulation, with coping involving regulatory processes that occur specifically in stressful contexts^5,10^. Here, we focus on coping strategies commonly employed by individuals in stressful situations.

It has been shown that the customary use of dysfunctional (or maladaptive) coping strategies, such as self-blame, is associated with higher levels of post-traumatic stress symptoms in students^11^. Similarly, Aldao et al. (2010) observed greater associations between maladaptive strategies (e.g., avoidance, rumination and suppression) and psychopathologies (e.g., anxiety and depression) in comparison to adaptive regulation strategies (e.g., problem solving, reappraisal, and acceptance)^12^. This raises the question of whether the presence of maladaptive strategies is more deleterious than the lack of adaptive coping strategies. A large body of research has focused on the effects of coping mechanisms on distinct physiological and psychological outcomes, such as physical functioning, perceived stress, anxiety and cortisol levels^13,14^. Here, we examined the effects of habitual coping styles (adaptive and maladaptive) on cardiovascular parameters (heart rate and heart rate variability) in response to a stressful task—oral speech presentation. Heart rate variability (HRV) can be defined as a noninvasive measure that reflects the oscillation of RR intervals due to sympathetic and parasympathetic influences in the sinoatrial node^15,16^. However, in short-term recording (e.g., 5 min) HRV is mainly influenced by the parasympathetic branch^17^. According to the neurovisceral integration model, it has been proposed that emotion regulation can be indexed by heart rate variability^17^, with higher values at rest usually associated with down regulation of negative affect, use of adaptive regulatory strategies and a more flexible emotional response^18^. In this model, the autonomic modulation of heart rate is influenced by brain areas (e.g., amygdala and prefrontal cortex), namely, the central autonomic network (CAN)^19^. The CAN allows the integration of adjusted physiological activation to adequate emotional responding imposed by situational demands. Thus, emotion regulation and coping processes are expected to be related to an individual's ability to adjust HRV on a momentary basis according to the circumstance^20^.

Studies that have elicited stress have typically observed an increase in heart rate (HR)^21,22^, demonstrating a predominance of the sympathetic axis or a decrease in parasympathetic activity (vagal withdrawal). However, the effects of stress induction on HRV changes are less clear, with some parameters showing increases or decreases in response to stress manipulations^23^. A reduction in HRV is observed in situations where strong stress responses are elicited^18,24–26^. For example, Souza et al. (2013) observed robust tachycardia and HRV reduction during stress induction (TSST) and a more efficient recovery in Brazilian peacekeepers with higher resilience trait and higher resting vagal tone^25^. On the other hand, a study from Schuber et al. (2009) demonstrated increases in heart rate and some HRV parameters (SDRR, HF and LF) during a short-term stress (speech task)^27^. Similarly, a recent study from Blons et al. (2019) reported increases in cardiac entropy (a non-linear marker of HRV) during a cognitive task representing self-regulatory effort. Importantly, when the task included additional stressors (e.g., examiners in the room), participants who showed an increase in anxiety levels exhibited a reduction in cardiac entropy^28^. This result points to the critical role of individual subjective differences in determining the direction of HRV changes in response to stress. In this context, the present study focused on the role of coping styles in HR and HRV changes in an oral speech task.

To our knowledge, there is no report in the literature of how the habitual use of coping strategies (coping styles) would modulate cardiac responses (HR and HRV changes) to a psychosocial stressor. Considering the sample as a whole, we expect to observe increases in HR and reductions in HRV parameters during the induction of the psychological stressor and a return of these parameters to baseline values in the recovery phase. However, when focusing on habitual coping strategies, since maladaptive strategies seem to be central in psychopathologies, our main hypothesis was that those participants who generally use more maladaptive strategies (highly maladaptive) on a daily basis would have greater reductions in HRV in response to the stress task and would fail to recover after the cessation of the stressor. Understanding whether differences in physiological responses are associated with coping strategies can be a key element to clarify the physiological changes underlying the psychopathological processes involving self-regulatory impairment (e.g., anxiety disorders).

## Methods

### Participants

A convenience sample of eighty-three undergraduate students from Universidade Federal Fluminense with a mean age of 20 years (SD ± 1.87) participated in the present study. As gender is a critical factor determining HRV, only females took part in the study^29,30^. All volunteers were recruited through advertising at university facilities and posts on social media. The exclusion criteria were being under pharmacological treatment (except contraceptives), and having a diagnosis of any medical condition that affects HRV (e.g., cardiovascular diseases). Six participants were excluded due to a respiration cycle outside the high-frequency (HF) band (0.15–0.4 Hz), eight for being a smoker, and nine for being above the cutoff value for obesity (BMI ≥ 30)^31^. In the final sample (n = 60) with a mean age of 20 years old (SD ± 1.77), height of 1.62 m (SD ± 0.06), weight of 59.60 kg (SD ± 10.38), and BMI of 22.61 (SD ± 3.55), 14 (23%) participants reported the use of oral contraceptives.

### Ethical statement

All participants provided written informed consent and all methods were carried out in accordance with relevant guidelines and regulations. The present study had formal approval from the Ethics Committee of the School of Medicine/Antônio Pedro University Hospital, Universidade Federal Fluminense. The present data were collected before the COVID-19 pandemic.

### Assessment instruments

#### Questionnaires

Coping style was assessed with the Brazilian version of the Brief COPE^7,32^. This inventory is a 28-item self-report scale comprising 14 conceptually different coping strategies that can be considered adaptive or maladaptive^7,33^. Items are rated on a 5-point Likert-scale format (0 to 4 points). Participants were instructed to complete the Brief COPE based on their usual ways of coping with stress, which occur on a daily basis.

The adaptive coping (total score range from 0 to 64) strategies encompass active coping (e.g., I have been concentrating my efforts on doing something about the situation I'm in), planning (e.g., I have been trying to come up with a strategy about what to do), instrumental support (e.g., I have been getting help and advice from other people), emotional support (e.g., I have been getting comfort and understanding from someone), positive reframing (e.g., I have been looking for something good in what is happening), religion (e.g., I have been praying or meditating), acceptance (e.g., I have been learning to live with it), and humor (e.g., I have been making fun of the situation). The maladaptive coping (total score range from 0 to 48) strategies consist of self-blame (e.g., I have been blaming myself for things that happened), denial (e.g., I have been refusing to believe that it has happened), venting (e.g., I have been expressing my negative feelings), behavioral disengagement (e.g., I have been giving up the attempt to cope), self-distraction (e.g., I have been turning to work or other activities to take my mind off things), and substance use (e.g., I have been using alcohol or other drugs to make myself feel better).

Finally, the high and low groups in the adaptive and maladaptive subscale were determined by calculating the median, with those above or equal to the median being considered high traits (e.g., high maladaptive or high adaptive) and those below considered low traits (low maladaptive or low adaptive).

#### Physiological measures

Electrocardiogram (ECG) signals were recorded using an ECG100C module coupled to a BIOPAC MP150 amplifier (BIOPAC System Inc.). All signals were digitized at a 1000 Hz sample rate. ECG signals were recorded using an adapted Lead I configuration (negative and positive electrodes attached to the chest). Participants were asked to remain seated during the whole experiment and to avoid movement to minimize artefacts in the signal.

Physiological data cleaning and analyses were performed in Kubios HRV software^34^. The software uses an in-house detection algorithm based on the Pan-Tompkins algorithm. Visual inspection of the raw ECG data was conducted to examine the presence of artifacts and an automatic artefact correction algorithm from Kubios HRV software was used. All data had less than 5% of artefacts. Heart rate and heart rate variability variables were quantified for each period of the experiment (rest, reactivity and recovery). HRV quantification followed the recommendations of the Task force of the European Society of Cardiology and North American Society of Pacing Electrophysiology^15^. For the time domain measures, we used the (1) standard deviation of the interbeat interval (IBI) of normal sinus beats (SDNN), which reflects all oscillatory components responsible for heart rate variability^16,17,35^ and the (2) root mean square of successive differences between normal heartbeats (RMSSD) to estimate the vagally mediated changes reflected in HRV^16,17,35^. For the power spectral density analyses, the Fast-Fourier transform (FFT) method was used to separate the HRV into its frequency components. There were two components considered in this study: (1) the high-frequency (HF) band, set at 0.15–0.4 Hz, which represents parasympathetic activity and is called the respiratory band because it corresponds to the HR variations related to the respiratory cycle and (2) the low-frequency (LF) band, set at 0.04–0.15 Hz, which may be produced by both the parasympathetic and sympathetic nervous systems and blood pressure regulation via baroreceptors, primarily by the PNS or by baroreflex activity alone. In resting conditions, the LF band reflects baroreflex activity and not sympathetic influences^35^. We used the algorithm of ECG-derived respiration (EDR) from Kubios HRV Premium to exclude those outside the HF band range (0.15–0.4 Hz).

All the HRV parameters used in the analyses were the natural log (ln) of the raw HRV values (ms or ms^2^). We reported the LF/HF ratio, HF n.u. and LF n.u. in Table 1, however, we did not perform any analysis with these parameters.

**Table 1:** Descriptive statistics of psychological and physiological variables.

	Mean (SD)	Min	Max	Skewness	Kurtosis
**Brief COPE**					
Maladaptive coping	23.07 (5.49)	10.00	35.00	0.04	− 0.31
Adaptive coping	36.90 (8.61)	17.00	55.00	− 0.01	− 0.25
**Rest**					
Heart rate (bpm)	88.33 (10.27)	67.05	110.44	− 0.10	− 0.16
EDR (Hz)	0.28 (0.05)	0.16	0.37	− 0.12	− 0.35
SDNN (ln)	3.56 (0.39)	2.57	4.81	0.42	1.89
SDNN (ms)	38.16 (18.07)	13.04	123.28	2.63	9.78
RMSSD (ln)	3.28 (0.52)	2.00	4.92	0.48	1.71
RMSSD (ms)	30.70 (20.96)	7.36	137.03	3.23	13.36
LF power (ln)	6.45 (0.75)	4.67	8.48	− 0.16	0.23
LF power (ms^2^)	833.31 (727.85)	106.91	4839.65	3.32	15.80
LF n.u	0.61 (0.17)	0.10	0.88	− 0.80	0.47
HF power (ln)	5.96 (1.06)	3.04	9.48	0.51	2.15
HF power (ms^2^)	808.27 (1839.41)	20.86	13,153.35	5.67	35.86
HF n.u	0.39 (0.17)	0.12	0.90	0.80	0.47
LF/HF ratio	2.17 (1.57)	0.11	7.56	1.23	1.45
**Reactivity**					
Heart rate (bpm)	96.22 (13.23)	68.83	128.68	0.14	− 0.59
EDR (Hz)	0.30 (0.05)	0.19	0.39	− 0.38	− 0.63
SDNN (ln)	3.45 (0.46)	2.34	4.77	0.46	0.92
SDNN (ms)	35.17 (19.08)	10.34	117.38	2.40	7.97
RMSSD (ln)	3.13 (0.60)	1.78	4.79	0.35	0.88
RMSSD (ms)	27.70 (21.34)	5.95	120.74	2.84	9.61
LF power (ln)	6.23 (0.94)	4.08	8.44	− 0.14	− 0.29
LF power (ms^2^)	769.71 (773.60)	59.07	4622.48	2.59	9.69
LF n.u	0.59 (0.17)	0.18	0.89	− 0.55	− 0.31
HF power (ln)	5.80 (1.18)	2.74	9.33	0.19	1.24
HF power (ms^2^)	736.80 (1604.87)	15.55	11,267.61	5.42	33.03
HF n.u	0.40 (0.17)	0.11	0.82	0.55	− 0.31
LF/HF ratio	2.04 (1.57)	0.22	8.27	1.68	3.57
**Recovery**					
Heart rate (bpm)	89.58 (10.05)	69.07	116.79	0.09	− 0.06
EDR (Hz)	0.27 (0.05)	0.15	0.38	− 0.15	− 0.45
SDNN (ln)	3.60 (0.40)	2.21	4.76	− 0.20	2.88
SDNN (ms)	39.66 (17.65)	9.13	116.53	2.37	8.74
RMSSD (ln)	3.25 (0.54)	1.57	4.80	0.14	2.18
RMSSD (ms)	30.13 (20.30)	4.81	121.37	2.98	11.30
LF power (ln)	6.57 (0.86)	4.11	8.22	− 0.55	0.33
LF power (ms^2^)	963.50 (765.31)	60.68	3707.17	1.63	3.11
LF n.u	0.66 (0.17)	0.24	0.92	− 0.59	− 0.48
HF power (ln)	5.78 (1.11)	2.47	9.10	0.16	1.74
HF power (ms^2^)	656.73 (1346.88)	11.81	8927.10	5.11	28.07
HF n.u	0.33 (0.17)	0.08	0.76	0.59	− 0.48
LF/HF ratio	2.96 (2.38)	0.32	11.45	1.59	2.74

*Bpm* beats per minute, *EDR* ECG-derived respiration, *SDNN* standard deviation of NN intervals. *RMSSD* Root mean square of successive RR interval differences, *LF power* absolute power of low frequency band heart rate variability, *HF power* absolute power of high frequency band heart rate variability, *n.u.* normalized units.

#### Study design and procedures

**Figure 1:**
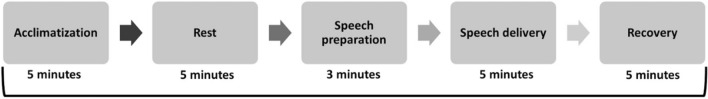
Experimental procedure.

Participants were asked not to eat or drink caffeine beverages 2 h before the appointment and to avoid moderate to intense physical activity on that day. We also asked them to not consume alcohol in the 24 h prior to the day of the experiment. All appointments were scheduled between 09 am and 07 pm. At the time of recruitment, the participants were not informed about the content of the task to avoid anticipation that could interfere with the stress response. Arriving at the laboratory, participants gave written consent to the experiment and answered a questionnaire with personal and medical information. Then, they were led to a temperature-controlled room (21–24 °C) where the ECG electrodes (Lead I) were placed on the chest to record the physiological signals during the entire experimental session. They were asked to remain seated in front of a computer where the task instructions would be given. After five minutes of acclimatization to the experimental room, the rest phase (baseline) was recorded for five minutes, where participants were instructed to stay relaxed and breathe spontaneously, without speaking and avoiding any movement. Immediately after the rest phase, instructions for the preparation phase were given. The task used here to induce acute moderate psychological stress was an oral speech task in which the participants had to prepare and deliver a speech to a camera^24,36^. Participants were informed that they had three minutes to mentally prepare a speech about the reasons they should be considered the best candidate for a dream job. An informative text on the computer screen also informed participants that the speech presentation was going to be recorded and then evaluated offline by three experimenters. After the preparation phase, participants delivered the speech to a camera that was in front of them for the next five minutes. Participants were instructed to keep talking through the entire speech phase (5 min). Finally, after the speech delivery, the participants were informed that the camera was no longer recording and that they could relax and should remain seated for five minutes for the recovery phase recording—see Fig. 1.

At the end of the recovery phase, it was revealed to the participant that the speech was not videotaped nor was it going to be evaluated. Then, after removing the physiological recording equipment, the participant responded to the psychometric scales. Since speech tasks can produce erratic respiration patterns and modulate HRV^37^, we did not use the speech delivery phase in the analyses. Thus, analyses were conducted using only three time points: rest (baseline), speech preparation (reactivity), and recovery.

### Statistical analyses

Descriptive statistics of the sample data were conducted (see Table 1). To test for normality, skewness and kurtosis were used (− 2 and 2). Heart rate variability data were natural log transformed for all statistical tests.

ANOVAs were used to assess the effects of coping styles on cardiovascular parameters in the stressful task. For each coping type (adaptive and maladaptive), factorial designs 3 × 2 (task phase: rest, reactivity and recovery x coping: high and low trait) were conducted for HR and each HRV parameter (SDNN, RMSSD, LF power, HF power). The Greenhouse–Geisser correction method was used to account for the lack of sphericity. For post hoc analysis, the adjusted p values with the Holm correction were used to account for alpha inflation. For the main effect of task phase, the correction was made for a family of 3 comparisons, and for the interactions between task x maladaptive coping (or adaptive coping), the correction was made for a family of 15 comparisons.

All the analyses and graphical representation were conducted in JASP^38^ and RStudio^39^. All tests were performed using a set level of significance of *p* > 0.05.

Power analysis was conducted in G*Power 3.1.9.4^40^ prior to the study. For a factorial 3 × 2 ANOVA with within-between interaction, a total sample size of 28 was necessary to achieve 80% power, considering an alpha of 0.05, effect size of 0.253 (η^2^partial of 0.06), and a correlation of 0.5 among the repeated measures.

## Results

### Task phase modulation of cardiac parameters

**Figure 2:**
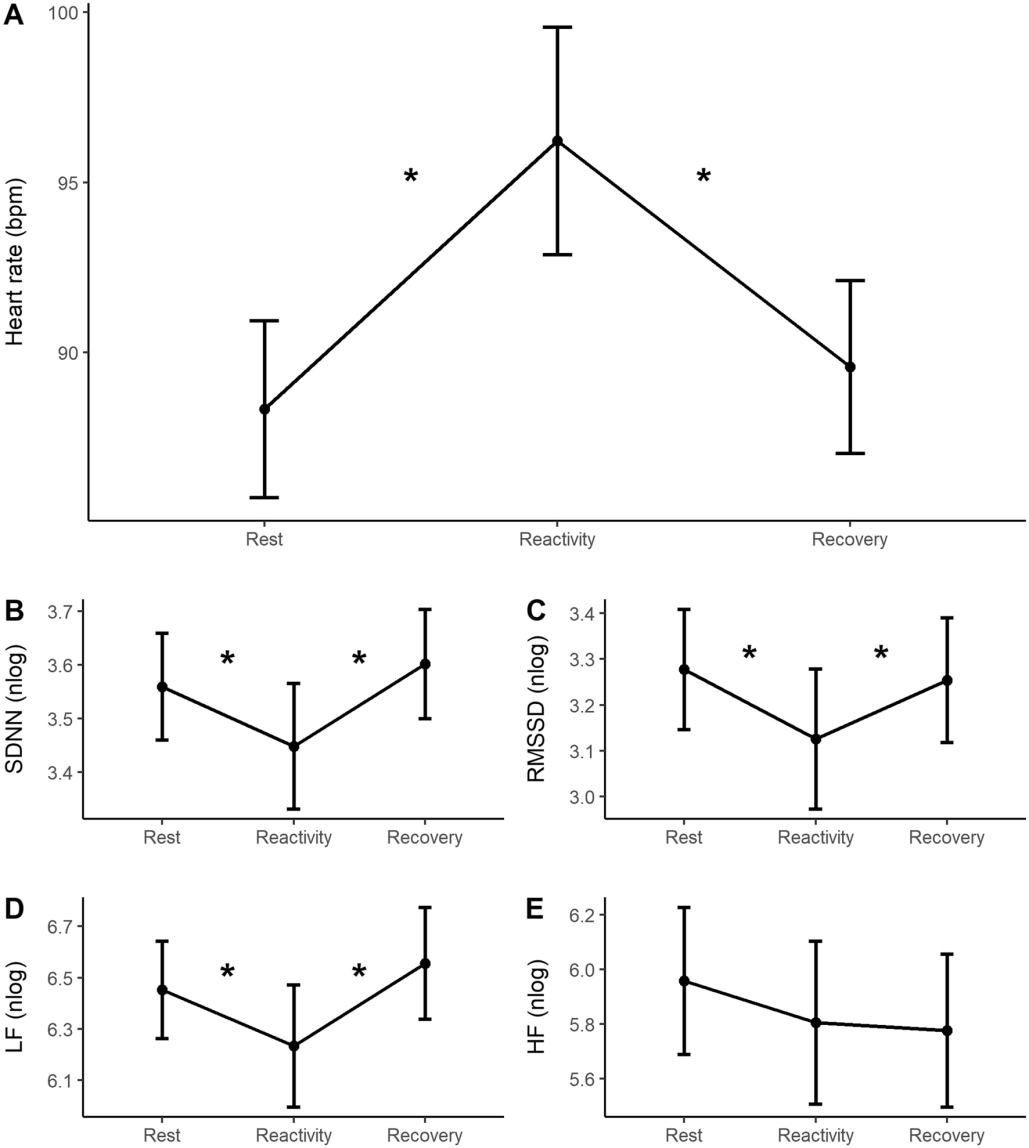
Mean and 95% CI (confidence interval) of the cardiac responses across task phases. (**A**) Heart rate; (**B**) SDNN; (**C**) RMSSD; (**D**) LF band; (**E**) HF band. *p ≤ 0.05.

HR (F(1.30, 77.13) = 42.82, *p* < 0.001), SDNN (F(1.53, 90.28) = 9.98, *p* < 0.001), RMSSD (F(1.46, 86.22) = 8.07, *p* = 0.002), and LF (F(2, 152) = 5.60, *p* = 0.005) were modulated by the task phase, and HF band did not change across the task phases (F(1.73, 102.12) = 2.43, *p* = 0.101)—see Fig. 2 and Supplementary Table 1 (Supplementary material). HR increased from rest (mean = 88.33) to reactivity (mean = 96.22, *p* < 0.001) and decreased from reactivity to the recovery phase (mean = 89.58, *p* < 0.001). SDNN decreased from the rest (mean = 3.56) to reactivity phase (mean = 3.44, p = 0.004) and increased from reactivity to the recovery phase (mean = 3.60, *p* < 0.001). RMSSD also had a reduction from rest (mean = 3.27) to the reactivity phase (mean = 3.13, *p* < 0.001) and increased from reactivity to the recovery phase (mean = 3.25, *p* = 0.004). Likewise, the LF band was reduced from the rest (mean = 6.45) to reactivity phase (mean = 6.23, *p* = 0.056) and increased from reactivity to the recovery phase (mean = 6.56, *p* = 0.004). Finally, as mentioned before, the HF band did not change across the task phases (*p* = 0.101).

The ANOVA and post hoc tables can be found in the Supplementary material.

### Interactions between task phase and adaptive coping on cardiac parameters

As shown in Supplementary Table 2 (Supplementary material), there was no interaction between adaptive coping and task phase for heart rate (F(1.30, 75.49) = 0.60, *p* = 0.482, η^2^partial = 0.010). No interaction was found between adaptive coping and task for SDNN (F(1.51, 87.81) = 0.73, *p* = 0.451, η^2^partial = 0.012), RMSSD (F(1.45, 84.02) = 0.43, *p* = 0.587, η^2^partial = 0.007), LF (F(2, 116) = 0.80, *p* = 0.451, η^2^partial = 0.014), and HF (F(1.71, 99.26) = 0.71, *p* = 0.471, η^2^partial = 0.012).

These results suggest that independent of the level of adaptive strategies employed daily by the participants, both low- and high-trait groups of adaptive coping have similar cardiac responses to a psychologically stressful situation.

### Interactions between task phase and maladaptive coping on cardiac parameters

Significant interactions were observed between task phase and group (high and low maladaptive) for time (SDNN and RMSSD) and frequency domain (LF) parameters of HRV. Although not significant, HF band had a strong tendency (*p* = 0.06).

**Figure 3:**
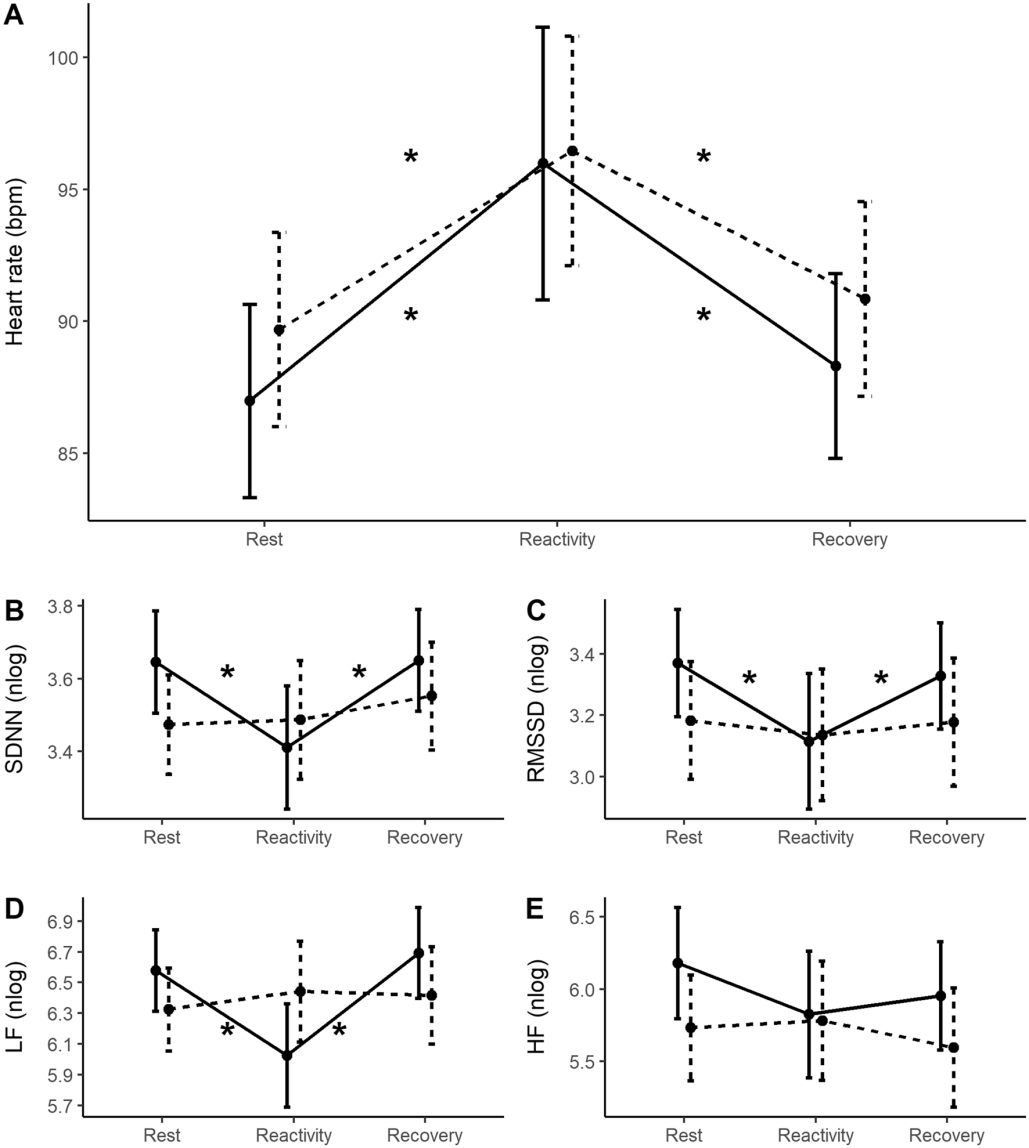
Mean and 95% CI (confidence interval) of the cardiac responses across task phases for the high (solid lines) and low maladaptive (dashed lines) groups. (**A**) Heart rate; (**B**) SDNN; (**C**) RMSSD; (**D**) LF; (**E**) HF. *p ≤ 0.05.

For HR—see Supplementary Table 3 (Supplementary material)—there was no interaction between task phase and maladaptive coping (F (1.31, 76.13) = 0.92, *p* = 0.365, η^2^partial = 0.016)—see Fig. 3A.

For SDNN, we observed an interaction between task phase and maladaptive coping group (F (1.59, 92.45) = 7.19, *p* = 0.003, η^2^partial = 0.110)—see Fig. 3B. More specifically, post hoc analyses revealed that only those with higher scores on maladaptive coping had a reduction in SDNN from rest (mean = 3.64) to the reactivity phase (mean = 3.41, *p* < 0.001) and then an increase from reactivity to the recovery phase (mean = 3.65, *p* < 0.001). There were no significant changes across the task phases for those with low scores on maladaptive strategies (rest mean = 3.47; reactivity mean = 3.49; recovery mean = 3.55; *p* = 1.00).

For RMSSD, there was a significant interaction between task phase and maladaptive coping (F (1.49, 86.48) = 3.96, *p* = 0.034, η^2^partial = 0.064)—see Fig. 3C. More specifically, like SDNN results, post hoc analyses revealed that only those with higher scores on maladaptive coping showed a reduction from rest (mean = 3.37) to the reactivity phase (mean = 3.11, *p* < 0.001) and an increase from reactivity to the recovery phase (mean = 3.33, *p* = 0.003). For those with lower scores on maladaptive coping there was no change across the task phase (rest mean = 3.18; reactivity mean = 3.14; recovery mean = 3.18; *p* = 1.00).

For the LF band, a significant interaction between task phase and maladaptive coping was found (F (2, 116) = 9.11, *p* < 0.001, η^2^partial = 0.136)—see Fig. 3D. Post hoc comparisons demonstrated that only those with higher scores on maladaptive coping showed a reduction in the LF band from rest (mean = 6.57) to the reactivity phase (mean = 6.02, *p* < 0.001) and then a increase from reactivity to the recovery phase (mean = 6.69, *p* < 0.001). There were no significant changes across the task phases for the low maladaptive coping group (rest mean = 6.33; reactivity mean = 6.44; recovery mean = 6.42; *p* = 1.00).

Finally, no interaction between task phase and maladaptive coping was found for the HF band. However, we found a strong tendency (F (1.76, 102.18) = 3.01, *p* = 0.060, η^2^partial = 0.049)—see Supplementary Table 3 and Fig. 3E. For this reason, we performed a post hoc analysis to observe if the HF band would present the same pattern of the previous results. The tests showed that only the high maladaptive group had a strong tendency of reduction from rest (mean = 6.18) to reactivity (mean = 5.83, *p* = 0.069) but not differing from reactivity to the recovery phase (mean = 5.95, *p* = 1.00). Again, the low maladaptive group had no significant changes across the task phases (rest mean = 5.73; reactivity mean = 5.78; recovery mean = 5.60; *p* = 1.00).

The ANOVA and post hoc tables can be found in the Supplementary material.

## Discussion

The present study investigated the effects of the habitual use of coping strategies on heart rate and heart rate variability changes in response to moderate acute stress (preparation for a speech). The psychosocial stress test induced an increase in heart rate and a reduction in heart rate variability for the whole sample. For heart rate, no differences between the high and low maladaptive or adaptive coping groups were found across the task phases. Regarding HRV, the results showed that females who typically used less maladaptive coping strategies did not exhibit a reduction in SDNN, RMSSD, HF and LF during the reactivity phase when compared to the rest phase. However, those females who often use more maladaptive strategies had significant reductions from rest to the reactivity phase for SDNN, RMSSD and LF band. Although not significant, the interaction between task phase and maladaptive coping for the HF band had a strong tendency. For the analyses with adaptive coping, we did not observe any interaction between task phase and the adaptive coping groups for any HRV parameter. In summary, only participants who often use more maladaptive strategies had significant reductions in HRV during the reactivity phase.

The increase in HR in both groups of maladaptive and adaptive coping levels (high and low) can be suggestive that independent of how one usually copes, the expected cardiac response (increase in heart rate) to a socially relevant stressful situation is present. In a similar study, Krkovic et al. (2018) evaluated salivary cortisol, heart rate, and other stress responses to an adapted version of the TSST (Trier Social Stress Test) and investigated how emotion regulation modulated this response^22^. Likewise, they did not find any association between maladaptive emotion regulation and heart rate response, suggesting that sympathetic activation dominance may be a hallmark in this widely employed psychological stressor, independent of individual differences. In fact, increases in heart rate during this type of acute stress are widely known through the literature on the preparation and delivery of speech^25,41–44^. Increases in heart rate have been found not only in this type of response but also in other physiological markers associated with the stress response. For example, Al’Absi et al. (1997) observed increases in heart rate, blood pressure, adrenocorticotropic hormone, and cortisol, as well as negative mood during a public speaking task, demonstrating that this kind of task is an appropriate experimental protocol to evoke a relatively high, stable, and homogenous response^44^. However, some individual differences do play a role in some of these stress response markers. For example, Mendonça-de-Souza et al. (2007) observed that only the unpleasant-primed group with high negative affect had significant increases in salivary cortisol concentration after an oral speech presentation. Those with low negative affect in the unpleasant-primed group and those with low or high negative affects in the pleasant-primed group had no significant changes in salivary cortisol^36^. Another study, which compared PTSD patients and trauma-exposed controls on their response to listening to their own traumatic script, showed an increase in HR for both groups. However, in contrast to PTSD patients, trauma-exposed controls did not show any significant changes in HRV at any point in the experimental procedure while the PTSD patient group had a sustained HRV reduction during the stressful task^45^.

The findings are in accordance with the pattern of results found in the present study: although HR changes were not affected by coping style, HRV changes were influenced by the high maladaptive group. Here, the results suggest that females with higher scores on maladaptive coping strategies had a reduction in time and frequency-domain parameters of HRV(SDNN, RMSSD, HF and LF power) when confronted by an acute psychological stressor. Interestingly, those with low maladaptive coping scores had no significant changes from rest to reactivity in those parameters, even though we observed a sympathetic predominance demonstrated by increases in heart rate in both groups. Thus, we can suggest that each group has distinct mechanisms underlying the heart rate increase during the acute stress phase. In the present study, SDNN exhibited no change from baseline to the reactivity phase for the low maladaptive group. Although considered a more general marker of variability, accounting for variation of different sources of physiological control mechanisms, in short-term resting recordings the primary source of the variation is parasympathetically-mediated respiratory sinus arrhythmia^17^. For RMSSD, which is widely used in the literature as a marker of parasympathetic activity^15–18,35^, we did not observe changes from rest to reactivity for the low maladaptive group. Regarding the frequency-domain, the power in the high and low-frequency component did not change from rest to reactivity for the low maladaptive group either, also suggesting a maintenance of vagal activity. As suggested by Reyes del Paso et al. (2013), although HRV components are primarily related to parasympathetic activity, each of these components is generated by different physiological mechanisms^46^. For example, HF power is related to respiratory influences (e.g., respiratory sinus arrhythmia), while LF power is associated with blood pressure control mechanisms (e.g., baroreflexes)^46,47^. Therefore, our results provide evidence for the parasympathetic involvement as a function of the habitual use of maladaptive coping styles. Critically, despite the expected tachycardia to acute stress, the parasympathetic system was relatively suppressed in participants habitually using more dysfunctional strategies and unaltered in those using less maladaptive strategies. It is important to cite Wilder’s law of the initial value, which could be another explanation of why the high maladaptive group had greater reductions in the reactivity phase^48^. When we observe the values of HRV, it seems that the high maladaptive group has higher HRV at baseline when compared to the low maladaptive group. However, the post hoc analyses showed no differences between high and low maladaptive group at baseline for all HRV parameters. Thereby, we propose that although both groups have a standard autonomic response to the mental stressor (HR increase), the low maladaptive group appears to maintain vagal activity (RMSSD, HF and LF) during the reactivity phase. In contrast, the high maladaptive group exhibited a reduction in HRV parameters during reactivity.

According to the neurovisceral integration model^49,50^, a set of neural structures (e.g., central autonomic network) involved in cognitive, affective, and autonomic regulation are related to HRV. The main idea is that cortical structures (e.g., prefrontal cortex) have an important role in the modulation of subcortical cardio-acceleratory circuits (e.g., amygdala) via an inhibitory pathway that is related to vagal function^50–52^. Knowing that many cognitive and affective processes depend upon structures like amygdala and prefrontal cortex, it is reasonable to think that there could exist a relationship between HRV and self-regulatory capabilities (e.g., coping strategies, emotion regulation).

It has been shown that low HRV at rest is related to poor coping styles (behavioral disengagement and denial) in bereaved individuals^53^, while high HRV at rest has been associated with greater use of engagement coping (e.g., seeking social support) and less probability of habitually employing disengagement coping strategies^54^. Regarding phasic HRV, some studies have observed influences from self-regulatory skills (e.g., emotion regulation). For example, Christou-Champi et al. (2015) found that after three training sessions practicing the use of cognitive reappraisal, the emotion regulation training group had phasic HRV (RMSSD) increases while reappraising unpleasant images^55^. Other studies cited in a review from Balzarotti et al. (2017) found increases in phasic HRV when participants were instructed to suppress or reappraise their emotions while discussing an upsetting topic with another participant^56^ or while viewing an anger-provoking film^57^. In addition, the modulation of phasic HRV by individual traits has also been demonstrated. For example, Di Simplicio et al. (2012) observed that those with high neuroticism had reductions in the HF band during cognitive reappraisal of negative stimuli (e.g., pictures), suggesting a loss of parasympathetic cardiovascular activity during emotion regulation as a function of neuroticism levels. In contrast, those with low neuroticism demonstrated increases in the HF band during emotion regulation (e.g., reappraisal) when compared to passive exposure to negative stimuli^58^. Interestingly, in the present study, those with low scores in maladaptive strategies had no significant changes in vagal activity (SDNN, RMSSD, HF and LF) across the task phases. As mentioned before, this could be interpreted as a maintenance of vagal activity during the stressful task and may be a marker of a successful regulatory effort to the task in hand. It is not clear whether the maintenance of parasympathetic activity in stressful contexts is adaptive. However, what is possible to gather from the literature is that HRV increases when participants are instructed to use regulation skills when viewing unpleasant pictures^55^, when individuals with low neuroticism reappraise negative stimuli^58^, or when participants with low anxiety perform a cognitive task with added stressors^28^.As low HRV at rest has been associated with anxiety, depression, increased risk of cardiovascular disease and mortality^59,60^, having reductions in HRV to every stressful event in our modern lives could be problematic in the long term. In fact, there is a long-standing hypothesis that suggests that cardiovascular hyperresponsiveness (e.g., heart rate, systolic and diastolic blood pressure) under stress is related to the development of cardiovascular diseases such as atherosclerosis and essential hypertension^61–65^.

There is no consensus in the literature about changes in the LF component in stressful circumstances, with some studies reporting increases while others reporting the opposite trend^23^. In a meta-analysis, Castaldo et al. (2015) only reported studies showing HRV changes to mental stressors without focusing on psychometric parameters (e.g., emotion regulation, coping skills, and anxiety symptoms)^23^. In our study, we observed reductions in time and frequency domain HRV parameters during the stress phase, as has been demonstrated in the majority of studies that focused on HRV changes to stress. However, when we consider the effects of habitual use of coping strategies, we showed that the HRV response to the stressor differs between those who habitually employ more or less maladaptive coping strategies. Another important consideration is that only a few studies have investigated the cardiac response to mental stress in females. For example, Adjei et al. (2018) found that during a mental math test, females had reductions in LFnu (normalized units), increases in HFnu (normalized units), and small increases in HR, while males had increases in LFnu, reductions in HFnu, and greater increases in HR, pointing to a different pattern of cardiac response to a mental stressor (math tests) as a function of gender^66^. The authors suggest that females exhibit a tend-and-befriend response to stress, employing social coping methods to combat stress, and this response is driven by the actions of estrogen and oxytocin, while males experience the conventional fight-or-flight response^66^. Finally, we highlight that, to our knowledge, this is the first study investigating how coping styles are associated with HRV changes to a psychological stress task in females.

The findings presented here add interesting evidence to the literature. However, there are some limitations. First, we did not assess a pure sympathetic measure (e.g., electrodermal activity) or a direct baroreflex measure. Second, no subjective measure of negative affect or perceived stress after each task phase was taken. Third, the menstrual cycle phase of the participants was not controlled (follicular or luteal phase). Lastly, the effects of the circadian rhythm on the HRV were not controlled either. However, due to the nature of the repeated measures analyses, in which we take measurements of the same participant in different time points, we do not expect this to be a critical factor in the conclusions of the present study.

Importantly, our data provides new evidence to the body of research assessing HRV responses to stressful tasks^23,26^. We can conclude that in this sample, females employing less maladaptive strategies had unaltered HRV (SDNN, RMSSD, HF and LF), depicting a different pattern of cardiac response. As discussed, the SDNN, RMSSD, HF and LF parameters in short-term recordings are mainly mediated by PNS, and this maintenance of baseline parasympathetic activity in a stressful context could be a sign of greater self-regulatory effort.

## Supplementary Information

Supplementary Information 1.

Supplementary Information 2.

## Data Availability

All data analyzed during this study are included in this published article (and its Supplementary Information files).
