# Supplementary material for: Association between distinct coping styles and heart rate variability changes to an acute psychosocial stress task
Source: Sci Rep. 2021 Dec 15;11:24025. doi: 10.1038/s41598-021-03386-6 (PMC8674249; doi:10.1038/s41598-021-03386-6)
Supplement: Supplementary file 2 — Supplementary Information 2. [file 41598_2021_3386_MOESM2_ESM.docx]

Association between distinct coping styles and heart rate variability changes to an acute psychosocial stress task

Arthur Viana Machado, Mirtes Garcia Pereira, Gabriela G. L. Souza, Mariana Xavier, Carolina Aguiar, Leticia de Oliveira, Izabela Mocaiber

# Supplementary material

**Supplementary table 1.** Main effects of task phase (n = 60).

|  | F (df, df residuals) | p-value | η^2^partial |
| --- | --- | --- | --- |
| **Heart Rate** |  |  |  |
| Task | 42.82 (1.30, 77.13) | **< 0.001** | 0.421 |
| **SDNN** |  |  |  |
| Task | 9.98 (1.53, 90.28) | **<0.001** | 0.145 |
| **RMSSD** |  |  |  |
| Task | 8.07 (1.46, 86.22) | **0.002** | 0.120 |
| **LF** |  |  |  |
| Task | 5.60 (2, 152) | **0.005** | 0.082 |
| **HF** |  |  |  |
| Task | 2.43 (1.73, 102.12) | 0.101 | 0.040 |

**Notes:** Df = Degrees of Freedom.

**Supplementary table 2.** Interaction effects between task phase and adaptive coping (n=60).

|  | F (df, df residuals) | p-value | η^2^partial |
| --- | --- | --- | --- |
| **Heart Rate** |  |  |  |
| Task x Adaptive | 0.60 (1.30, 75.49) | 0.482 | 0.010 |
| **SDNN** |  |  |  |
| Task x Adaptive | 0.73 (1.51, 87.81) | 0.451 | 0.012 |
| **RMSSD** |  |  |  |
| Task x Adaptive | 0.43 (1.45, 84.02) | 0.587 | 0.007 |
| **LF** |  |  |  |
| Task x Adaptive | 0.80 (2, 116) | 0.451 | 0.014 |
| **HF** |  |  |  |
| Task x Adaptive | 0.71 (1.71, 99.26) | 0.700 | 0.004 |

**Notes:** Df = Degrees of Freedom.

**Supplementary table 3.** Interaction effects between task phase and maladaptive coping (n=60).

|  | F (df, df residuals) | p-value | η^2^partial |
| --- | --- | --- | --- |
| **Heart Rate** |  |  |  |
| Task x Maladaptive | 0.92 (1.31, 76.13) | 0.365 | 0.016 |
| **SDNN** |  |  |  |
| Task x Maladaptive | 7.20 (1.59, 92.45) | **0.003** | 0.110 |
| **RMSSD** |  |  |  |
| Task x Maladaptive | 3.96 (1.49, 86.48) | **0.034** | 0.064 |
| **LF** |  |  |  |
| Task x Maladaptive | 9.11 (2, 116) | **<0.001** | 0.136 |
| **HF** |  |  |  |
| Task x Maladaptive | 3.01 (1.76, 102.18) | **0.060** | 0.049 |

**Notes:** Df = Degrees of Freedom.

**Supplementary table 4.** Post hoc analyses for heart rate main effect of task phase.

|  |  | Mean Difference | SE | p-value |
| --- | --- | --- | --- | --- |
| rest | reactivity | -7.887 | 0.916 | **<0.001** |
|  | recovery | -1.245 | 0.916 | 0.177 |
| reactivity | recovery | 6.643 | 0.916 | **<0.001** |

**Notes:** Holm’s adjusted p-values for compating a family of 3. SE = Standard Error.

**Supplementary table 5.** Post hoc analyses for SDNN main effect of task phase.

|  |  | Mean Difference | SE | p-value |
| --- | --- | --- | --- | --- |
| rest | reactivity | 0.111 | 0.035 | **0.004** |
|  | recovery | -0.042 | 0.035 | 0.239 |
| reactivity | recovery | -0.153 | 0.035 | **<0.001** |

**Notes:** Holm’s adjusted p-values for compating a family of 3. SE = Standard Error.

**Supplementary table 6.** Post hoc analyses for RMSDD main effect of task phase.

|  |  | Mean Difference | SE | p-value |
| --- | --- | --- | --- | --- |
| rest | reactivity | 0.152 | 0.041 | **<0.001** |
|  | recovery | 0.023 | 0.041 | 0.570 |
| reactivity | recovery | -0.128 | 0.041 | **0.004** |

**Notes:** Holm’s adjusted p-values for compating a family of 3. SE = Standard Error.

**Supplementary table 7.** Post hoc analyses for LF main effect of task phase.

|  |  | Mean Difference | SE | p-value |
| --- | --- | --- | --- | --- |
| rest | reactivity | 0.219 | 0.098 | **0.056** |
|  | recovery | -0.103 | 0.098 | 0.297 |
| reactivity | recovery | -0.322 | 0.098 | **0.004** |

**Notes:** Holm’s adjusted p-values for compating a family of 3. SE = Standard Error

**Supplementary table 8.** Post hoc analyses for SDNN interaction between maladaptive coping and task phase.

|  |  | Mean Difference | SE | p-value |
| --- | --- | --- | --- | --- |
| high, maladaptive, rest | low, maladaptive, rest | 0.172 | 0.108 | 1.000 |
|  | high, maladaptive, reactivity | 0.235 | 0.048 | **<0.001** |
|  | low, maladaptive, reactivity | 0.159 | 0.108 | 1.000 |
|  | high, maladaptive, recovery | -0.005 | 0.048 | 1.000 |
|  | low, maladaptive, recovery | 0.093 | 0.108 | 1.000 |
| low, maladaptive, rest | high, maladaptive, reactivity | 0.063 | 0.108 | 1.000 |
|  | low, maladaptive, reactivity | -0.013 | 0.048 | 1.000 |
|  | high, maladaptive, recovery | -0.177 | 0.108 | 1.000 |
|  | low, maladaptive, recovery | -0.079 | 0.048 | 1.000 |
| high, maladaptive, reactivity | low, maladaptive, reactivity | -0.076 | 0.108 | 1.000 |
|  | high, maladaptive, recovery | -0.240 | 0.048 | **<0.001** |
|  | low, maladaptive, recovery | -0.142 | 0.108 | 1.000 |
| low, maladaptive, reactivity | high, maladaptive, recovery | -0.164 | 0.108 | 1.000 |
|  | low, maladaptive, recovery | -0.066 | 0.048 | 1.000 |
| high, maladaptive, recovery | low, maladaptive, recovery | 0.098 | 0.108 | 1.000 |

**Notes:** Holm’s adjusted p-values for compating a family of 15. SE = Standard Error.

**Supplementary table 9.** Post hoc analyses for RMSSD interaction between maladaptive coping and task phase.

|  |  | Mean Difference | SE | p-value |
| --- | --- | --- | --- | --- |
| high, maladaptive, rest | low, maladaptive, rest | 0.188 | 0.143 | 1.000 |
|  | high, maladaptive, reactivity | 0.256 | 0.056 | **<0.001** |
|  | low, maladaptive, reactivity | 0.235 | 0.143 | 1.000 |
|  | high, maladaptive, recovery | 0.042 | 0.056 | 1.000 |
|  | low, maladaptive, recovery | 0.192 | 0.143 | 1.000 |
| low, maladaptive, rest | high, maladaptive, reactivity | 0.068 | 0.143 | 1.000 |
|  | low, maladaptive, reactivity | 0.047 | 0.056 | 1.000 |
|  | high, maladaptive, recovery | -0.146 | 0.143 | 1.000 |
|  | low, maladaptive, recovery | 0.005 | 0.056 | 1.000 |
| high, maladaptive, reactivity | low, maladaptive, reactivity | -0.021 | 0.143 | 1.000 |
|  | high, maladaptive, recovery | -0.214 | 0.056 | **0.003** |
|  | low, maladaptive, recovery | -0.064 | 0.143 | 1.000 |
| low, maladaptive, reactivity | high, maladaptive, recovery | -0.193 | 0.143 | 1.000 |
|  | low, maladaptive, recovery | -0.042 | 0.056 | 1.000 |
| high, maladaptive, recovery | low, maladaptive, recovery | 0.151 | 0.143 | 1.000 |

**Notes:** Holm’s adjusted p-values for compating a family of 15. SE = Standard Error.

**Supplementary table 10.** Post hoc analyses for LF interaction between maladaptive coping and task phase.

|  |  | Mean Difference | SE | p-value |
| --- | --- | --- | --- | --- |
| high, maladaptive, rest | low, maladaptive, rest | 0.253 | 0.219 | 1.000 |
|  | high, maladaptive, reactivity | 0.553 | 0.130 | **<0.001** |
|  | low, maladaptive, reactivity | 0.137 | 0.219 | 1.000 |
|  | high, maladaptive, recovery | -0.114 | 0.130 | 1.000 |
|  | low, maladaptive, recovery | 0.161 | 0.219 | 1.000 |
| low, maladaptive, rest | high, maladaptive, reactivity | 0.301 | 0.219 | 1.000 |
|  | low, maladaptive, reactivity | -0.116 | 0.130 | 1.000 |
|  | high, maladaptive, recovery | -0.367 | 0.219 | 1.000 |
|  | low, maladaptive, recovery | -0.091 | 0.130 | 1.000 |
| high, maladaptive, reactivity | low, maladaptive, reactivity | -0.416 | 0.219 | 0.785 |
|  | high, maladaptive, recovery | -0.668 | 0.130 | **<0.001** |
|  | low, maladaptive, recovery | -0.392 | 0.219 | 0.921 |
| low, maladaptive, reactivity | high, maladaptive, recovery | -0.251 | 0.219 | 1.000 |
|  | low, maladaptive, recovery | 0.024 | 0.130 | 1.000 |
| high, maladaptive, recovery | low, maladaptive, recovery | 0.276 | 0.219 | 1.000 |

**Notes:** Holm’s adjusted p-values for compating a family of 15. SE = Standard Error.

**Supplementary table 11.** Post hoc analyses for HF interaction between maladaptive coping and task phase.

|  |  | Mean Difference | SE | p-value |
| --- | --- | --- | --- | --- |
| high, maladaptive, rest | low, maladaptive, rest | 0.448 | 0.288 | 1.000 |
|  | high, maladaptive, reactivity | 0.355 | 0.123 | **0.069** |
|  | low, maladaptive, reactivity | 0.397 | 0.288 | 1.000 |
|  | high, maladaptive, recovery | 0.226 | 0.123 | 0.887 |
|  | low, maladaptive, recovery | 0.585 | 0.288 | 0.640 |
| low, maladaptive, rest | high, maladaptive, reactivity | -0.093 | 0.288 | 1.000 |
|  | low, maladaptive, reactivity | -0.051 | 0.123 | 1.000 |
|  | high, maladaptive, recovery | -0.222 | 0.288 | 1.000 |
|  | low, maladaptive, recovery | 0.137 | 0.123 | 1.000 |
| high, maladaptive, reactivity | low, maladaptive, reactivity | 0.042 | 0.288 | 1.000 |
|  | high, maladaptive, recovery | -0.129 | 0.123 | 1.000 |
|  | low, maladaptive, recovery | 0.230 | 0.288 | 1.000 |
| low, maladaptive, reactivity | high, maladaptive, recovery | -0.171 | 0.288 | 1.000 |
|  | low, maladaptive, recovery | 0.188 | 0.123 | 1.000 |
| high, maladaptive, recovery | low, maladaptive, recovery | 0.359 | 0.288 | 1.000 |

**Notes:** Holm’s adjusted p-values for compating a family of 15. SE = Standard Error.


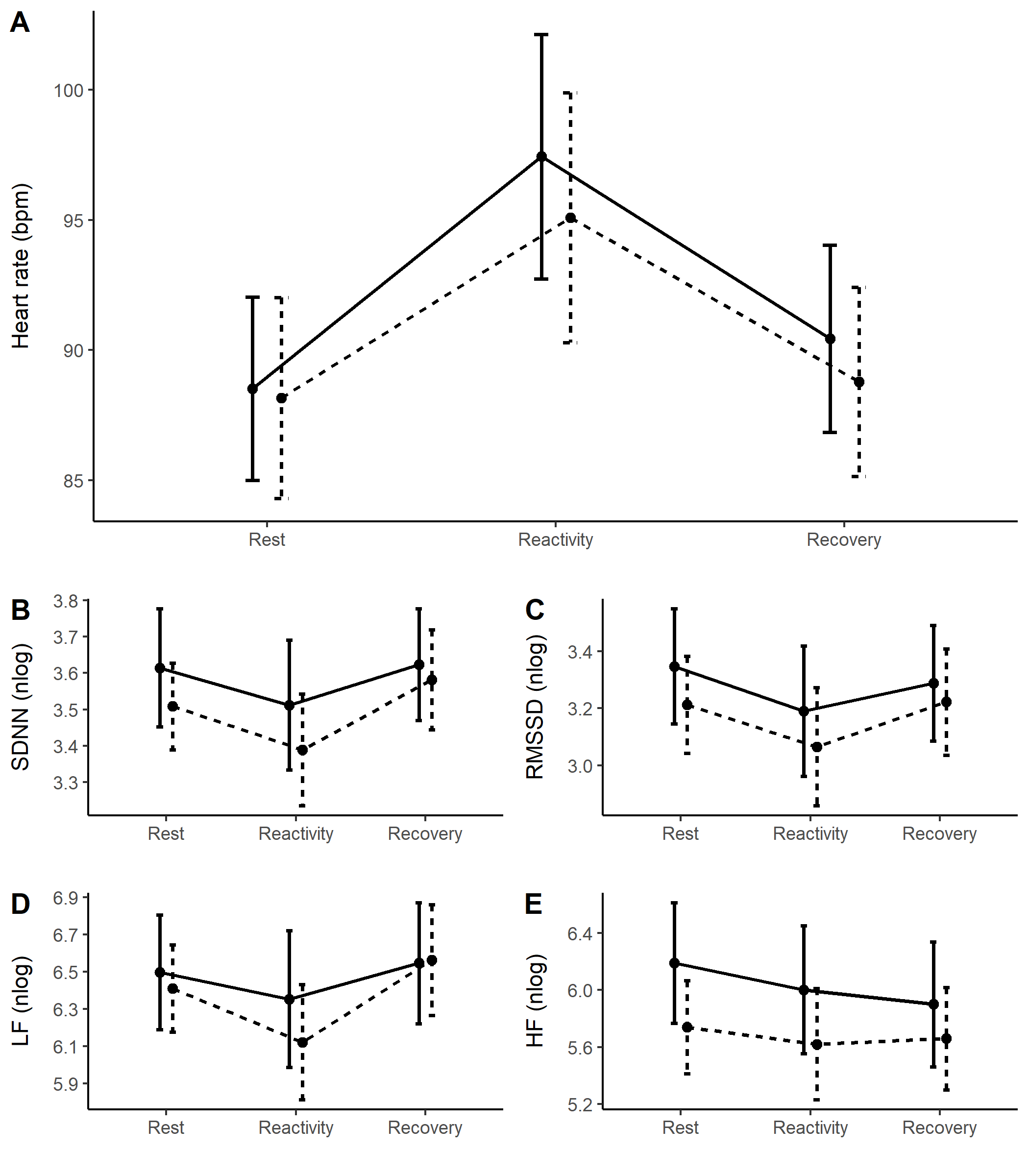
**Supplementary figure 1.** Mean and 95% CI (confidence interval) of the cardiac responses across task phases for the high (solid lines) and low adaptive (dashed lines) groups. (A) Heart rate; (B) SDNN; (C) RMSSD; (D) LF; (E) HF. *p ≤ 0.05
